# MADS-Box Gene Classification in Angiosperms by Clustering and Machine Learning Approaches

**DOI:** 10.3389/fgene.2018.00707

**Published:** 2019-01-08

**Authors:** Yu-Ting Chen, Chi-Chang Chang, Chi-Wei Chen, Kuan-Chun Chen, Yen-Wei Chu

**Affiliations:** ^1^Institute of Genomics and Bioinformatics, National Chung Hsing University, Taichung, Taiwan; ^2^Ph.D. Program in Medical Biotechnology, National Chung Hsing University, Taichung, Taiwan; ^3^School of Medical Informatics, Chung-Shan Medical University, Taichung, Taiwan; ^4^IT Office, Chung Shan Medical University Hospital, Taichung, Taiwan; ^5^Department of Computer Science and Engineering, National Chung-Hsing University, Taichung, Taiwan; ^6^Biotechnology Center, Agricultural Biotechnology Center, Institute of Molecular Biology, National Chung Hsing University, Taichung, Taiwan

**Keywords:** ABCDE model, MADS-box gene, phylogenetic tree, support vector machine, machine learning

## Abstract

The MADS-box gene family is an important transcription factor family involved in floral organogenesis. The previously proposed ABCDE model suggests that different floral organ identities are controlled by various combinations of classes of MADS-box genes. The five-class ABCDE model cannot cover all the species of angiosperms, especially the orchid. Thus, we developed a two-stage approach for MADS-box gene classification to advance the study of floral organogenesis of angiosperms. First, eight classes of reference datasets (A, AGL6, B12, B34, BPI, C, D, and E) were curated and clustered by phylogenetic analysis and unsupervised learning, and they were confirmed by the literature. Second, feature selection and multiple prediction models were curated according to sequence similarity and the characteristics of the MADS-box gene domain using support vector machines. Compared with the BindN and COILS features, the local BLAST model yielded the best accuracy. For performance evaluation, the accuracy of *Phalaenopsis aphrodite* MADS-box gene classification was 93.3%, which is higher than 86.7% of our previous classification prediction tool, iMADS. Phylogenetic tree construction – the most common method for gene classification yields classification errors and is time-consuming for analysis of massive, multi-species, or incomplete sequences. In this regard, our new system can also confirm the classification errors of all the random selection that were incorrectly classified by phylogenetic tree analysis. Our model constitutes a reliable and efficient MADS-box gene classification system for angiosperms.

## Introduction

Angiosperms, i.e., flowering plants, have evolved a most remarkable flower to ensure fertilization and reproduction. Indeed, angiosperms have evolved many specialized flowering processes to adapt to a wide range of environments as well as to attract animals, which help facilitate their reproduction. Thus, angiosperms comprise a diverse group of plants, accounting for ∼80% of all plant species (Christenhusz and Byng, 2016), and they constitute the source materials for the production of many foods, drugs, wood, paper, and fiber.

Studies of the model plant Arabidopsis indicate that dicotyledonous flowers contain four organs, namely the sepal, petal, stamen, and carpels, which are located on four concentric whorls. Flowering processes and floral organ determination are controlled by MADS-box genes (Theissen et al., 2000). The encoded proteins share a MADS (M) domain at the N-terminus, which is a conserved 56–amino acid residue region that is named for the initials of four members of this family: MCM1, AG, DEF, and SRF (Yang et al., 2012). The MADS-box genes have been classified as type I and type II on the basis of phylogenetic analysis (Alvarez-Buylla et al., 2000). Type I, named the M-type, contains the conserved M domain and the large variability region at the C-terminus (Masiero et al., 2011). Type II is known as MIKC-type, which contains and is named for the M domain, intervening (I) domain, keratin-like (K) domain, and C-terminal (C) domain (Kaufmann et al., 2005). Functionally, the M domain has the DNA binding activity, the I domain influences the DNA-binding dimerization, and the K domain can form amphipathic helices that mediate dimerization of MADS-box proteins and also are involved in the formation of other complexes (Egea-Cortines et al., 1999; Yang et al., 2003). The C domain, which is the most variable in sequence and function, is involved in transcriptional activation and formation of higher-order transcription factor complexes and also contributes to MADS-box protein interaction specificity (van Dijk et al., 2010; Callens et al., 2018). In plants, M-type MADS-box genes are involved in reproduction, especially female gametophyte, embryo, and endosperm development (Masiero et al., 2011), and MIKC-type MADS-box genes participate in meristem differentiation, flowering, fruit development, and the determination of floral organ identity according to the ABCDE model (Callens et al., 2018).

**FIGURE 1:**
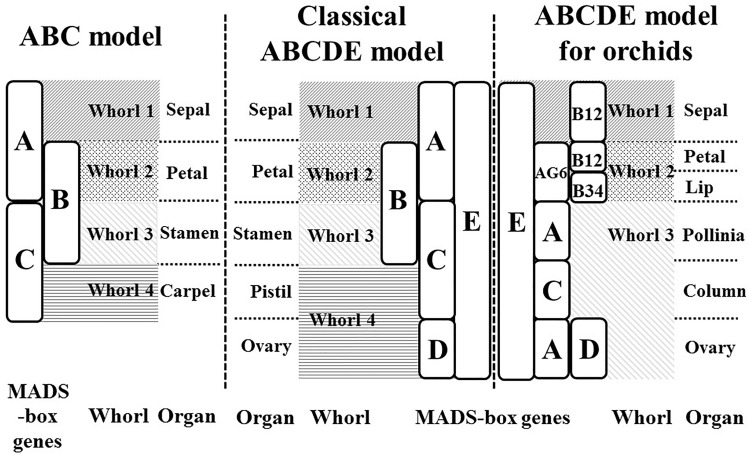
ABC and ABCDE models. The flowering models show the MADS-box genes which are responsible for flower organ development in the different floral whorl.

The ABCDE model, which originated from the ABC model (Coen and Meyerowitz, 1991), explains the genetic mechanisms of flower development and floral organ identification through the complex interaction of MIKC-type MADS-box genes (Figure 1). On the basis of their homeotic functions, the floral organ identity MADS-box genes have been divided into A, B, C, D, and E classes (Theissen, 2001). A- and E-class proteins are together responsible for sepal development in the first floral whorl, the combination of A-, B-, and E-class proteins specifically controls petal formation in the second whorl, the combination of B-, C-, and E-class proteins regulates stamen differentiation in the third whorl, the combination of C- and E-class proteins specifies carpel development in the fourth whorl, and the combination of D- and E-class proteins is required for ovule identity (Murai, 2013; Figure 1). Mutant phenotype analysis has facilitated cloning and identification of many MADS-box genes. The A-class genes include *APELATA 1 (AP1)* and *FRUITFULL (FUL)* (Gu et al., 1998); the B-class genes include *APELATA 3 (AP3)*, *PISTILLATA (PI)*, and *GLOBOSA (GLO)* (Zahn et al., 2005); the C-class gene is *AGAMOUS (AG)* (Mizukami and Ma, 1997); the D-class genes include *SEEDSTICK (STK)* and *SHATTERPROOF1 (SHP1)* (Favaro et al., 2003); and the E-class genes include *SEPALLATA1 (SEP1)*, *SEP2*, *SEP3*, and *SEP4* (Ditta et al., 2004). Phylogenetic analysis is currently the most common method for MADS-box gene classification. Because of massive, multi-species, and incomplete sequences, phylogenetic tree construction can be time-consuming and result in classification errors (Su et al., 2013a). Incomplete assembly sequences are becoming more abundant with the widespread use of next-generation sequencing. Thus, the use of phylogenetic analysis for MADS-box gene classification is increasingly challenging.

In our previous study, we used a machine-learning approach to construct a MADS-box gene classification prediction tool, iMADS (Yang et al., 2012). This tool reduces the time required to generate output prediction and presents reliable and systematic results to users. However, there are still some deficiencies to conquer. First, the training dataset needs to be updated and filtered more precisely. Second, the training model of iMADS was constructed on the basis cross-alignment of whole sequences, which results in a lower performance on partial sequence prediction. Third, some plants that contain unique floral organs do not fit the ABCDE model. For example, the flowers of Orchidaceae, one of the most diverse and widespread horticultural plants, contain six floral organs: the sepal in whorl 1, the petal and lip in whorl 2, and the pollinia, column, and pedicel in whorl 3 (Su et al., 2013b). The classical ABCDE model cannot explain the determination of orchid-specific flower organs, the lip and pollinia. According to the transcriptomic sequences, microarray analysis, and quantitative PCR validation, Su et al. established a modified ABCDE model extended to eight classes to explain how the lip and pollinia are determined (Su et al., 2013b; Figure 1). In this modified model, in addition to C, D, and E classes, the A-class genes are divided into AP1 (A) and AGL6 classes, and the B-class genes are grouped into AP3-1,2 (B12), AP3-2,4 (B34), and PI (BPI). In *Phalaenopsis aphrodite*, the differentiation of the sepal requires AGL6, B12, BPI, and E-class genes, the petal requires AGL6, B12, B34, BPI, and E class genes, the lip requires AGL6, B34, BPI, and E-class genes, the pollinia require A and BPI class genes, the column requires AGL6, B34, BPI, C and E-class genes, and the pedicel requires A, AGL6, BPI, C, D, and E-class genes. On the basis of their study, we constructed a new automatic MADS-box genes classification platform to encompass more species of angiosperms.

In this study, we incorporated MADS-box genes of orchids into the training dataset to improve the performance of classification. The training model of the system occurs in two stages. To account for unique flower organs, the MADS-box genes are divided into eight classes rather than the five classes of the original ABCDE model. In addition, to test various features, we constructed multiple prediction models according to domain characteristics using support vector machines (SVMs). From the independent and error classification of phylogenetic results, we found that using the domain database as the training model and using BLAST as the feature yielded the best accuracy. In brief, this system can analyze different lengths of query sequences and automatically optimizes the prediction result corresponding to the extended eight-class model.

## Materials and Methods

**FIGURE 2:**
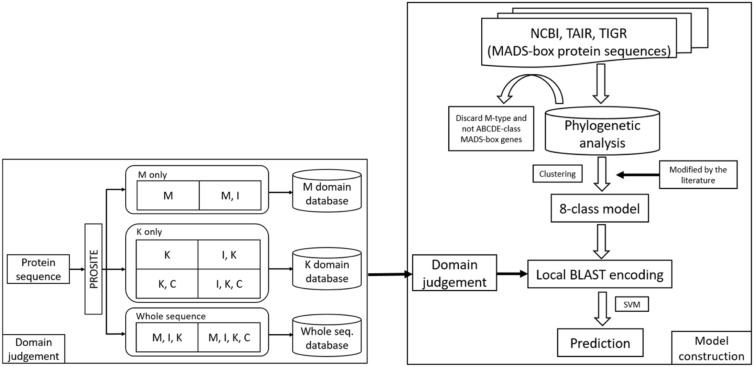
Flowchart of the MADS-box gene classification system. The MADS-box genes were collected from NCBI, TAIR, and TIGR (right). After phylogenetic analysis, only MIKC-type genes were selected and clustered into eight class training models. To avoid errors, all the classification was verified in the literature. For domain judgement, the query sequence was assessed by PROSITE to determine whether it was a MIKC-type gene and to identify its domain content (left), and then the sequence was sent to create features for classification.

In this study, we constructed the classification models for MADS-box genes using SVMs. In brief, the MADS-box genes were collected from NCBI, TAIR, and TIGR by key word search. The collection included M- and MIKC-type genes. Among them, only the MIKC-type genes, which involve the determination of floral organ identity, were selected and further classified into eight classes as a training dataset by unsupervised algorithms. According to the characteristics of the structure, the whole sequence and the four domains were used to create features via BLAST (Altschul et al., 1990), COILS (Lupas et al., 1991), and BindN (Wang and Brown, 2006) as input to LIBSVM (Fan et al., 2005) to construct the classification models. To improve the accuracy, we used PROSITE to determine whether the query sequence was a MIKC-type gene and to identify its domain content, and then the sequence was sent to create features for classification. The flowchart of the MADS-box gene classification system is shown in Figure 2, and the details of model construction are described as follows.

### Data Collection and Filtration

All the MADS-box protein sequences, including M- and MIKC-type genes, were collected from different flowering plants. For the training dataset curation, 89 *Arabidopsis* sequences were obtained from “The Arabidopsis Information Resource” (TAIR, http://www.arabidopsis.org) (Huala et al., 2001), 76 *Oryza* sequences were obtained from The Rice Genome Annotation (TIGR^1^) (Yuan et al., 2003), 47 orchid sequences (except *Phalaenopsis equestris* and *Oncidium*) were obtained from The National Center for Biotechnology Information (NCBI^2^) and 4 monocot plant sequences (*Lilium longiflorum*, LMADS_2, LMADS_10; *Hyacinthus orientalis*, HOMADS_1; *Agapanthus praecox*, APMADS2) were also from NCBI (Table 1) (216 in total). Combining the MUSCLE alignment tool and construction of a phylogenetic tree (using the neighbor-joining method and running 1000 bootstraps) of Molecular Evolutionary Genetics Analysis (MEGA 5.2) (Tamura et al., 2011) and information from the literature, the 216 sequences were divided into two groups: 133 MIKC-type sequences and 83 of M-type sequences. Some MIKC-type genes, which are involved in root development (ANR1; Gan et al., 2005) or regulate flowering time (SOC1; Moon et al., 2003), were discarded. Only the genes that directly regulate floral organs were filtered as the training dataset. Ultimately, 85 MIKC-type genes were further selected and classified into eight classes: 13 sequences of A, 9 sequences of B12, 10 sequences of B34, 10 sequences of BPI, 14 sequences of C, 8 sequences of D, 14 sequences of E, and 7 sequences of AGL6. To establish the independent testing datasets, 15 MADS-box genes of *P. aphrodite* and 11 of *Oncidium* Gower Ramsey were obtained from Orchidstra 2.0^3^ (Supplementary Datasets).

**Table 1:** Whole data collection.

Species	MADS-box genes No.	M-type gene No.	MIKC-type gene No.	Training dataset No.	Data source
*Arabidopsis*	89	49	40	16	TAIR^a^
*Oryza*	76	34	42	17	TIGR^b^
*Paphiopedilum*	6	0	6	6	NCBI^c^
*Phalaenopsis equestris*	14	0	14	14	NCBI^c^
*Dendrobium*	20	0	20	20	NCBI^c^
*Cymbidium*	7	0	7	7	NCBI^c^
*Lilium longiflorum*	2	0	2	2	NCBI^c^
*Hyacinthus orientalis*	1	0	1	1	NCBI^c^
*Agapanthus praecox*	1	0	1	1	NCBI^c^
*P. equestris*	15	0	15	0	Orchidstra 2.0^d^
*Oncidium*	11	0	11	0	Orchidstra 2.0^d^


*^a^TAIR, The Arabidopsis Information Resource, http://www.arabidopsis.org.*

*^b^TIGR, The Rice Genome Annotation, http://rice.plantbiology.msu.edu.*

*^c^NCBI, The National Center for Biotechnology Information, http://www.ncbi.nlm.nih.gov.*

*^d^For independent testing dataset construction, the MADS-box genes of P.equestris and Oncidium Gower Ramsey were obtained from Orchidstra 2.0 (http://orchidstra2.abrc.sinica.edu.tw).*

### Feature Encoding

**FIGURE 3:**
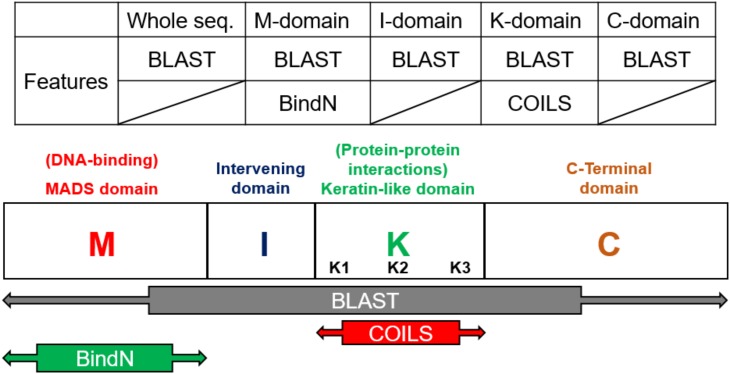
Feature selection according to domain-specific characteristics. On the basis of sequence conservation within structures, the whole sequence and four domains were used independently to create five features via BLAST. BindN was created according to the DNA-binding function of the M domain, and COILS was created according to the coiled-coils structure of the K domain.

According the characteristics of the structure, the models were constructed on the basis of the four domains and the whole sequences of MIKC-type genes. The prediction tools, Basic Local Alignment Search Tool (BLAST) (Altschul et al., 1990) and BindN (Wang and Brown, 2006), used as features by protein sequences, and COILS (Lupas et al., 1991), used as features by secondary protein structures, were used to identify the contribution to classification (Figure 3). In general, the whole sequences and the four MIKC domains individually used BLAST as features. Additionally, the M domain used BindN for encoding because it is the main DNA-binding region, and the K domain used COILS for encoding because it is the key region for coiled-coil structure formation. Finally, all of the features were integrated into machine learning by the SVM format. All the features are described as follows (Figure 3).

#### BLAST

**FIGURE 4:**
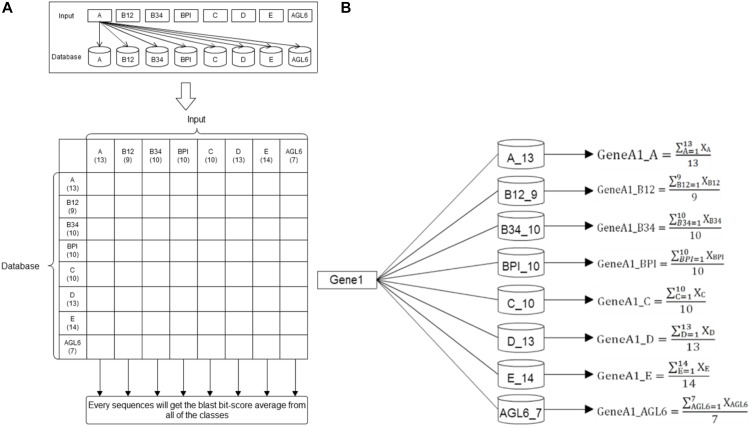
BLAST methodflowchart. **(A)** The training datasets. Here 86 reference sequences were classified into eight training datasets. Every reference sequence was inputted to compare with other sequences within the same class and to count the average BLAST score of this class. Then, this sequence was compared with other sequences of the other seven datasets to count their specific averages as the same flow. The number under the class designation represents the number of sequences within each dataset. **(B)** Basic Local Alignment Search Tool. After individually comparing all the reference sequences in the eight databases, the average BLAST scores were counted and presented as eight features for each input.

Basic local alignment search tool compares protein sequences to databases, calculates the statistical significance of matches, and finds regions of similarity between biological sequences (Altschul et al., 1990). In this study, we used the standalone version of BLAST to get the average *p*-value from the pairwise comparison of the request protein sequence and 8-class model sets. The eight average *p*-value will be encoded as features for SVM training. The eight training databases—A, B12, B34, BPI, C, D, E, and AGL6 – were constructed in our system. According the characteristics of the structure, the whole sequence and four domains were independently used to create five features via BLAST (Figure 3). The query sequence was compared with all the sequences in the database. For any e-values less than 10^-5^, the BLAST feature was encoded as the average of bi-scores. If all the e-values were less than 10^-5^, the BLAST feature was encoded as 0. Compared with eight databases individually, we obtained eight features for each input (Figure 4).

#### BindN

BindN is a bioinformatics tool for predicting DNA- or RNA-binding residues in amino acid sequences (Wang and Brown, 2006). The M domain of MADS-box genes, which contains a helix-loop-helix super-secondary structure related to DNA binding and is involved in dimerization, was used to create a feature by BindN.

#### COILS

COILS is a program that compares a sequence to a database of coiled-coils, derives a similarity score, and then calculates the probability of the coiled-coil formation (Lupas et al., 1991). The K domain of MIKC-type MADS-box proteins can form the leucine-zipper super-secondary structure (a short coiled-coil of two parallel helices), which is critical for dimerization. The two parallel helices are formed by several (abcdefg)_n_ heptad repeats. Among them, hydrophobic amino acids usually exist in the “a” and “d” positions, which cause the K domain to form a series of amphipathic α-helices and dimerize with other protein via hydrophobic interactions. In this study, we used a MTIDK matrix with weighting (weights: a,d = 2.5 and b,c,e,f,g = 1.0), and combined with 3-window width for feature encoding.

### LIBSVM

Among machine learning algorithms, the SVM, a supervised learning model with associated learning algorithms that analyze data and recognize patterns, is used for classification and regression analysis. LIBSVM is a library for SVMs. It incorporates software for support vector classification, regression, and distribution estimation, and supports multi-class classification (Fan et al., 2005). In this study, we used BLAST, COILS, and BindN features to build up nine training models to help classify the unknown-class MADS-box genes into one of the eight classes.

### Motif Discovery and Annotation in the C Domain

Compared with the other domains, the C domain has the lowest sequence conservation and most diverse function, which may improve the degree of computing discrimination. This region might exist in specific structures to achieve different functions. We selected parts of classes that could easily lead to confusion in classification: **“**Group B”, including B12, B34 and BPI classes; “Group AE”, including A, E, and AGL6; and “Group CD”, including C and D. Focused on these three groups, we used MEME (Bailey et al., 2015) and TOMTOM (Gupta et al., 2007) to find the novel motifs and to annotate their functions using the JASPAR plant motif database (Khan et al., 2018).

## Results and Discussion

### Model Construction and Validation

**FIGURE 5:**
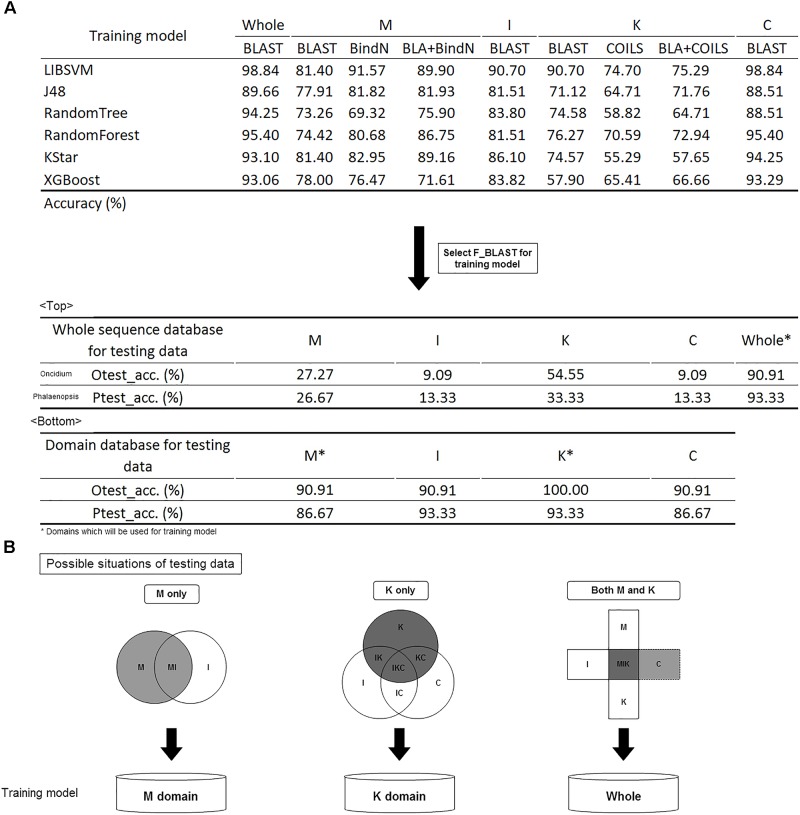
Model construction and validation. **(A)** The accuracy of the different training models. Comparison of different tools; BLAST was selected for future evaluation of the effects of whole sequences and domain database training sets. **(B)** Filtration system to screen the domain content of the input and to subject it to the suitable model for classification prediction.

Before constructing the classification model, it was necessary to make a tool comparison among BLAST, COILS, and BindN which was used along or combined with other on whole or domain’s sequence analysis in different training models. The six classification algorithms of LIBSVM, RandomForest, J48, RandomTree, KStar and XGBoost were used for model selection (Hall et al., 2009; Qiang et al., 2018; Wei et al., 2018). The trained predictor was evaluated with 3-fold cross-validation, and its corresponding accuracy is shown in Figure 5A. In brief, BLAST expressed the highest accuracy with whole sequences and I, K, and C domains, but BindN had the best accuracy with the M domain. That is, the significance of DNA/RNA-binding residues could improve the classification. The highly conserved coiled-coil formation in the K domain caused the lowest discriminating rate by COILS.

Given its higher accuracy in most models, BLAST was selected for the training model. Because of the numerous bursts of sequence generation in the post-genome era, the number of incomplete sequences are increasing. To identify the effects of whole and partial gene sequences on this classification system, we used MADS-box genes of *P. equestris* and *Oncidium* Gower Ramsey to establish a whole sequence database and a domain database as independent testing sets. Compared with the whole sequence database, we found that this classification system expressed much higher accuracy in the domain database on incomplete sequence classification (Figure 5A). For example, if the input was a MADS-box genes of *P. equestris* containing only the M domain, the corresponding accuracy of the whole sequence database and the domain database was 27.27 and 90.91%, respectively. Thus, we constructed a filtration system to screen the domain content of the input and then subject it to the suitable model for classification prediction (Figure 5B).

### Performance of Independent Dataset

Using LIBSVM to perform predictions, the internal statistical analysis function can be used to calculate the probability that all data are predicted to be in any category. Of the prediction results of the testing data set, 24 of 26 were classified correctly (92.31%) (Tables 2, 3). The *Oncidium* MADS-box gene *OMADS3* had similar probabilities to be B12 or B34 (0.35 and 0.36, respectively) and was incorrectly predicted as class B34 (Table 2). The *P. aphrodite* MADS-box gene *PATC052371* has a expression pattern similar to class C, but was predicted as class D in this study (Table 3). Although *PATC052371* was deleted in the updated Orchidstra 2.0 database (Chao et al., 2017), that could be attributed to missed annotation. Thus, we can ignore this classification error.

**Table 2:** Performance of *Oncidium* Gower Ramsey independent dataset.

Gene	Gene class	Predicted class	Confidence score							
			A	B12	B34	BPI	C	D	E	AGL6
OMADS10	A	A	0.71	0.02	0.03	0.03	0.03	0.05	0.09	0.05
OMADS3	B12	B34^∗^	0.06	0.35	0.36	0.09	0.02	0.04	0.04	0.04
OMADS5	B12	B12	0.03	0.76	0.08	0.04	0.02	0.02	0.02	0.03
OMADS9	B34	B34	0.03	0.09	0.77	0.04	0.01	0.02	0.02	0.02
OMADS8	BPI	BPI	0.05	0.04	0.07	0.70	0.02	0.03	0.04	0.04
OMADS4	C	C	0.04	0.02	0.03	0.02	0.78	0.05	0.04	0.03
OMADS2	D	D	0.04	0.02	0.02	0.02	0.06	0.79	0.03	0.03
OMADS6	E	E	0.03	0.01	0.02	0.02	0.02	0.03	0.82	0.06
OMADS11	E	E	0.06	0.01	0.02	0.02	0.02	0.04	0.74	0.08
OMADS1	AGL6	AGL6	0.11	0.03	0.04	0.05	0.04	0.06	0.15	0.54
OMADS7	AGL6	AGL6	0.05	0.02	0.03	0.03	0.03	0.05	0.08	0.71


*^∗^OMADS3 was incorrectly classified as B34.*

**Table 3:** Performance of *P. aphrodite* independent dataset.

Gene	Gene class	Predicted class	Confidence score							
			A	B12	B34	BPI	C	D	E	AGL6
PATC145405	A	A	0.65	0.02	0.04	0.04	0.03	0.04	0.11	0.06
PATC154931	A	A	0.73	0.02	0.03	0.03	0.02	0.04	0.07	0.05
PATC133864	B12	B12	0.07	0.39	0.31	0.09	0.02	0.04	0.04	0.04
PATC138350	B34	B34	0.03	0.11	0.72	0.04	0.02	0.02	0.03	0.03
PATC154853	B34	B34	0.03	0.08	0.77	0.04	0.01	0.02	0.02	0.03
PATC152852	BPI	BPI	0.05	0.04	0.06	0.71	0.02	0.03	0.04	0.04
PATC138585	C	C	0.03	0.02	0.02	0.02	0.82	0.03	0.03	0.03
PATC052371	D^∗^	D^∗^	0.02	0.01	0.02	0.02	0.06	0.83	0.02	0.02
PATC155109	C	C	0.03	0.02	0.02	0.02	0.80	0.05	0.03	0.03
PATC202120	D	D	0.05	0.02	0.03	0.03	0.07	0.74	0.04	0.04
PATC138540	E	E	0.04	0.01	0.02	0.02	0.02	0.03	0.81	0.06
PATC141808	E	E	0.07	0.02	0.02	0.03	0.03	0.04	0.73	0.07
PATC152066	E	E	0.04	0.01	0.02	0.02	0.02	0.03	0.81	0.06
PATC154379	AGL6	AGL6	0.06	0.02	0.03	0.03	0.03	0.04	0.08	0.70
PATC138772	AGL6	AGL6	0.10	0.03	0.04	0.04	0.03	0.05	0.14	0.57


*^∗^PATC052371 was incorrectly predicted as class D in this study, but it was deleted in the updated Orchidstra 2.0 database.*

### Performance Comparison With the iMADS Classification Method

We previously constructed iMADS, which is a bioinformatics tool for classification of Angiosperm MADS-box genes (Yang et al., 2012). We used the same features of sequence similarity in this prediction tool. However, we divided the MADS-box genes into eight classes rather than five classes in iMADS, and analyzed the domain content of the input before subjecting it to the suitable model for classification prediction in this study. *P. aphrodite* MADS-box genes were selected for performance comparison. As shown in Table 4, the C-class *PATC052371AGL6* was incorrectly predicted as D-class by both systems. The D-class *PATC202120* was correctly classified in this study, but was incorrectly predicted as C-class by iMADS. The accuracy of this classification system (93.33%) is higher than that of iMADS (86.67%).

**Table 4:** Performance comparison of classification methods using *P. aphrodite* MADS-box genes.

Gene	iMADS			This study		
	5-class	Predicted class	T/N	8-class	Predicted class	T/N
PATC145405_A	A	A	T	A	A	T
PATC154931_A	A	A	T	A	A	T
PATC154379_AGL6	A/E	E	T	AGL6	AGL6	T
PATC138772_AGL6	A/E	E	T	AGL6	AGL6	T
PATC133864_B12	B	B	T	B12	B12	T
PATC138350_B34	B	B	T	B34	B34	T
PATC154853_B34	B	B	T	B34	B34	T
PATC152852_BPI	B	B	T	BPI	BPI	T
PATC052371_C	C	D	N^∗^	C	D	N^∗^
PATC138585_C	C	C	T	C	C	T
PATC155109_C	C	C	T	C	C	T
PATC202120_D	D	C	N^∗^	D	D	T
PATC138540_E	E	E	T	E	E	T
PATC141808_E	E	E	T	E	E	T
PATC152066_E	E	E	T	E	E	T
Accuracy (%)	86.67%			93.33%		


*^∗^Incorrect classification.*

Phylogenetic analysis has been the most common method for MADS-box gene classification. Among 226 MADS-box genes, there were 16 classification errors on the tree. Of these, 5 *Picea abies* B-class genes were incorrectly grouped into E-class by phylogenetic analysis. Because these genes are derived from gymnosperms rather than angiosperms, we selected the other 11 genes for performance evaluation of the prediction tools. As shown in Table 5, iMADS corrected most classification errors (72.73%) but not for *ZMM1*, *SHP1* and *SHP2*. The resolution between C and D-class was poor for iMADS. This new classification system can correct all the classification errors by phylogenetic analysis and provide a reliable classification approach.

**Table 5:** Performance comparison of classification methods using classification error of the phylogenetic tree.

Pro_ID^a^	Gene name	Class	Predicted class		
			Phylogenetic tree	iMADS	This study
91207151	OsMADS20	A	E	A	A
602900	Silene latifolia_SLM1	C	A/E	C	C
887579	Rumex acetosa_RAP1	C	A/E	C	C
2981131	Populus trichocarpa_PTAG1	C	A/E	C	C
1001935	ZMM1	D	C	C^∗^	D
1345505	Arabidopsis thaliana_AG	C	D	C	C
57157565	AVAG2	D	C	D	D
41387778	Eustoma grandiflorum_MADS1	D	C	D	D
52548060	SHP1	D	C	C^∗^	D
52548152	SHP2	D	C	C^∗^	D
42794574	Meliosma dilleniifolia_MdAG2	D	C	D	D
Accuracy				72.73%	100%


*^a^NCBI Protein_ID.*

*^∗^Incorrect classification by iMADS.*

### The Correlation Between Conservation and Prediction Accuracy of the Four Domains

To determine the variant accuracy of the four domain prediction models, we used the pairwise distance method (Tamura et al., 2011) to identify the correlation between conservation and prediction accuracy (Table 6). The pairwise distance method calculates the pairwise distance average from total scores by constructing a matrix comparing sequences with each other. The domain with a higher pairwise distance average represents its higher diversity. Combining the resulting pairwise distance and prediction accuracy, we found that the diversity in the four domains from high to low was C, I, K, and M which was positively correlated with the accuracy. Thus, when the diversity of a domain was lower, the prediction was more likely to be incorrect.

**Table 6:** Comparison the classification performance and conservation of the four domains.

	M	I	K	C
Accuracy (%)	81.395	90.700	90.700	98.837
Distance average (variance)	0.285	0.679	0.609	0.854
Standard error estimate (S.E.)	0.036	0.055	0.029	0.008


### Novel Coding Motif Analysis in the C Domain

**FIGURE 6:**
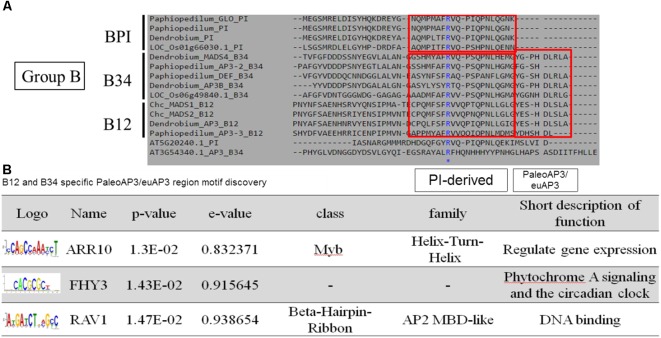
Novel coding motif investigation and annotation of the C domains of Group B genes. **(A)** Comparison of the C domains of Group B genes. The universal PI-derived motif was found in all Group B genes, whereas the paleoAP3/euAP3 motif was found only in B12 and B34. **(B)** Novel motif identification of the paleoAP3/euAP3 region. The coding DNA sequences of paleoAP3/euAP3 regions were subjected to MEME analysis, and ARR10, FHY3, and RAV1 motifs were then identified and annotated.

According to the pairwise distance and prediction accuracy results, the C domain expressed the highest diversity and best discrimination rate among the four domains. This indicated that there could be some specific structures in C domains that correspond to different classes of MADS-box genes. A comparison of the MADS-box genes belonging to Group B indicated that all of them contain a PI-derived motif, whereas B12 and B34 have an additional paleoAP3/euAP3 motif (Figure 6A). MEME and TOMTOM analyses revealed three coding motifs – ARR10, FHY3, and RAV1 – in the C domains of B12 and B34 (Figure 6B). ARR10, a helix-turn-helix super-secondary family motif, serves as a two-component response regulator involving a His-to-Asp phosphate signal transduction system (Hosoda et al., 2002). RAV1, a DNA-binding motif, exists in almost all eight classes and may be a universal key tool of MADS-box genes (Kagaya et al., 1999). The FHY3 motif involves transcriptional regulation of phytochrome A signaling and the circadian clock (Li et al., 2011). The FHY3 motif exists in B12 and B34 (APETALA3), but not BPI (PISTILLATA), which may reflect their diverse functions (Lamb and Irish, 2003).

**FIGURE 7:**
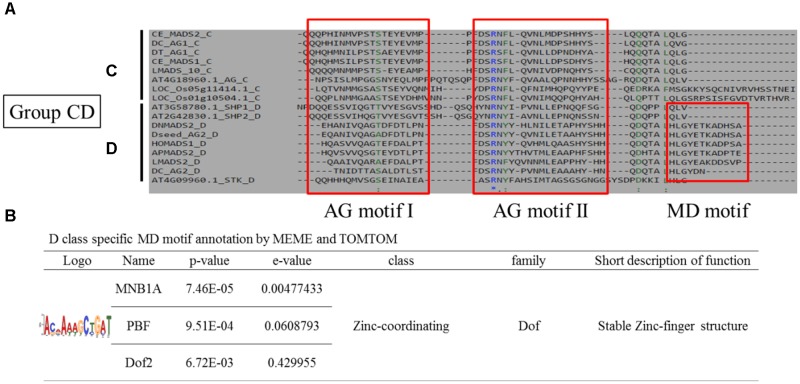
Novel coding motif investigation and annotation of the C domains of Group CD genes. **(A)** Comparison of the C domains of Group CD genes. Two universal motifs, AG motifs I and II, were found in Group C and D genes, whereas the MD motif was found only in Group D. **(B)** Novel motif identification of the MD region. The coding DNA sequences of MD regions were subjected to MEME analysis and MNB1A, PBF, and Dof2 motif were then identified and annotated.

Upon comparing the MADS-box genes belonging to Group CD, we found that they all share AG motif I and AG motif II motifs close to the middle region of the C terminal, but a specific MD motif is found in the C terminal of Group D genes (Figure 7A). We further investigated three functional coding motifs – MNB1A, PBF, and Dof2 – in this region by MEME and TOMTOM analyses (Figure 7B). These motifs all belong to the Dof gene family, a family of transcription factors, and may form a single zinc finger for DNA recognition (Yanagisawa and Schmidt, 1999). A protein–protein interaction structure, the coiled-coil, also exists in the zinc-finger of Dof, and thus we predict that MNB1A, PBF, and Dof2 motifs may involve an interaction with Group E genes. There was no significant motif identified in the Group AE genes.

### Tissue-Specific Coding Motif Analysis of MADS-Box Genes Among Multiple Species

DNA motifs are short, conserved functional regions. They are presumed to be involved in RNA localization, translation efficacy, mRNA splicing, mRNA stability, and accessibility to the translation machinery (Ding et al., 2012). The structure specificity could also indicate their key functionality. We collected MADS-box gene sequences from different species and used JASPAR to individually annotate their coding motifs (Supplementary Tables S1–S4). Class-unique motifs represent the unique motif in a particular class, such as SEP3 in Arabidopsis class A genes, PIF5 in class B, SOC1 in class D, and TGA1 in class E (Supplementary Table S1). These motifs could relate to the unique functions of the particular class. Tissue-related motifs indicated that the motifs exist in the genes expressed in a particular tissue. We found several tissue-specific motifs, such as SOC1 in carpels of Arabidopsis, bZIP910 in lodicules of rice, SPE3 in lips of *P.* *aphrodite*, and HAT5 in carpels of *Oncidium* (Supplementary Tables S1–S4). We also found some organism-specific motifs; the myb.Ph3 motif is unique to the MADS-box genes of Arabidopsis, whereas abi4, ERF1, and Gamyb are only found in rice. The specific motifs could be used for prediction or classification reference.

## Conclusion

Phylogenetic analysis usually contains classification errors and is time-consuming for massive, multi-species, or incomplete sequences. To solve this problem, we used machine learning approaches to establish a reliable and efficient MADS-box gene classification system for angiosperms. This classification system analyzes the domain content and then automatically subjects the query sequence to a suitable BLAST model. Corresponding to the extended eight classes, this classification system can also correct almost all the incorrect classifications generated from phylogenetic tree analysis. We also identified several class-specific, tissue-specific, and organism-specific coding motifs to use for classification or as future functional investigation references.

## Author Contributions

Y-TC, C-CC, and Y-WC conceived the study and drafted the manuscript. C-WC and K-CC collected the datasets and created the work-flow.

## Conflict of Interest Statement

The authors declare that the research was conducted in the absence of any commercial or financial relationships that could be construed as a potential conflict of interest.

## Footnotes

1 http://rice.plantbiology.msu.edu

2 http://www.ncbi.nlm.nih.gov

3 http://orchidstra2.abrc.sinica.edu.tw
