# Supplementary material for: MADS-Box Gene Classification in Angiosperms by Clustering and Machine Learning Approaches
Source: Front Genet. 2019 Jan 8;9:707. doi: 10.3389/fgene.2018.00707 (PMC6333052; doi:10.3389/fgene.2018.00707)
Supplement: Supplementary file 1 [file Table_1.docx]

Supplementary Material

MADS-Box Gene Classification in Angiosperms by Clustering and Machine Learning Approaches

Yu-Ting Chen^1,2#^, Chi-Chang Chang^3,4#^, Chi-Wei Chen^1,5^, Kuan-Chun Chen^1^, Yen-Wei Chu^1,2,6*^

^1^Institute of Genomics and Bioinformatics, National Chung Hsing University, 145 Xingda Rd., South District, Taichung City 402, Taiwan

^2^ Ph.D. Program in Medical Biotechnology, National Chung Hsing University, Taichung, Taiwan

^3^ School of Medical Informatics, Chung-Shan Medical University, Taichung, Taiwan

^4^ IT Office, Chung Shan Medical University Hospital, Taichung, Taiwan

^5^ Department of Computer Science and Engineering, National Chung-Hsing University, 145 Xingda Rd., South District, Taichung 402, Taiwan;

^6^ Biotechnology Center, Agricultural Biotechnology Center, Institute of Molecular Biology, National Chung Hsing University, 145 Xingda Rd., South District, Taichung 402, Taiwan

#Contribution equally.

*** Correspondence:** Corresponding Author: ywchu@nchu.edu.tw

**Keywords: ABCDE model, MADS-box gene, phylogenetic tree, support vector machine, machine learning**

# Table S1 Tissue-related coding motifs analysis of Arabidopsis MADS-box genes

| **Class** | **Sepal** | **Petal** | **Stamen** | **Carpel** | **Class-unique  motifs** | **Essential for each class and tissue** |
| --- | --- | --- | --- | --- | --- | --- |
| **A** | HMG-1, SEP3 | |  |  | SEP3 | ARR10  Dof2  Dof3  MNB1A  PBF |
| **AGL6** | HMG-1, myb.Ph3 | | | | ND^a^ |  |
| **B** |  | bZIP910, TGA1A, PIF5 | |  | PIF5 |  |
| **C** |  |  | HMG-1, HAT5 | | ND^a^ |  |
| **D** |  |  |  | HMG-1, bZIP910, TGA1A, SOC1 | SOC1 |  |
| **E** | HMG-1, bZIP910, TGA1A, TGA1, myb.Ph3, HAT5 | | | | TGA1 |  |
| **Tissue-related**  **motifs** | HMG-1  TGA1A  bZIP910  myb.Ph3  SEP3  TGA1  HAT5 | HMG-1  TGA1A  bZIP910  myb.Ph3  PIF5  SEP3  HAT5  TGA1 | HMG-1  TGA1A  bZIP910  myb.Ph3  HAT5  PIF5  TGA1 | HMG-1  TGA1A  bZIP910  myb.Ph3  SOC1^b^  HAT5  TGA1 |  |  |

Relative profile score threshold = 95%

^a^ No significant motif was found.

^b^ Organ-specific motif exits in MADS-box genes.

**Table S2 Tissue-related coding motifs analysis of rice MADS-box genes**

| **Class** | **Glume** | **Lodicule** | **Stamen** | **Carpel** | **Class-unique  motifs** | **Essential for each class and tissue** |
| --- | --- | --- | --- | --- | --- | --- |
| **A** | ERF1, abi4, bZIP910, TGA1A | |  |  | ND^a^ | Dof2  Dof3  PBF  MNB1A  HMG-1  ARR10 |
| **AGL6** | ERF1 | |  | ERF1 | ND^a^ |  |
| **B** |  | ERF1, PIF4, abi4, PIF5, CDC5, TGA1A, SMZ, bZIP910, PIF3 | |  | SMZ, PIF3, PIF4, PIF5 |  |
| **C** |  |  | ERF1, abi4 | | ND^a^ |  |
| **D** |  |  |  | ERF1, abi4 | ND^a^ |  |
| **E** | ERF1, abi4, TGA1A, bZIP910, Gamyb | | | | Gamyb |  |
| **Tissue-related**  **motifs** | bZIP10  TGA1A  ERF1  abi4  Gamyb | TGA1A  bZIP910^b^  ERF1  abi4  CDC5  SMZ  PIF3  PIF4  PIF5  Gamyb | bZIP10  TGA1A  ERF1  abi4  CDC5  SMZ  PIF3  PIF4  PIF5  Gamyb | ERF1  bZIP10  TGA1A  abi4  Gamyb |  |  |

Relative profile score threshold = 95%

^a^ No significant motif was found.

^b^ Organ-specific motif exits in MADS-box genes.

**Table S3 Tissue-related coding motifs analysis of *P. aphrodite* MADS-box genes**

| **Class** | **Sepal** | **Petal** | **Lip** | **Pollinia** | **Column** | **Ovary** | **Class-unique  motifs** | **Essential for each class and tissue** |
| --- | --- | --- | --- | --- | --- | --- | --- | --- |
| **A** |  |  |  | HMG-1, bZIP910, TGA1A, ARR10, PI |  | HMG-1, bZIP910, TGA1A, ARR10, PI | PI | Dof2  Dof3  MNB1A  PBF |
| **AGL6** | HMG-1, ARR10, TGA1A | | | | | | ND^a^ |  |
| **PATC154379 (lip-specific)** |  |  | HMG-1, ARR10, SEP3 |  |  |  | SEP3 |  |
| **B12** | ARR10 | |  |  |  |  | ND^a^ |  |
| **B34** |  | SMZ, ARR10, TGA1, TGA1A, bZIP910,  HMG-1 | |  |  |  | SMZ |  |
| **BPI** | ARR10 | | | | | | ND^a^ |  |
| **C** |  |  |  |  | ARR10, HMG-1, ABI3 | | ABI3 |  |
| **D** |  |  |  |  |  | HMG-1 | ND^a^ |  |
| **E** | HMG-1, ARR10, PIF3, PIF5, HAT5 | | |  | HMG-1, ARR10, PIF3, PIF5, HAT5 | | PIF3, PIF5, HAT5 |  |
| **Tissue-related**  **motifs** | ARR10  HMG-1  TGA1A  PIF3  PIF5  HAT5 | ARR10  HMG-1  TGA1A  bZIP910  TGA1  PIF3  PIF5  HAT5  SMZ | ARR10  HMG-1  TGA1A  bZIP910  TGA1  PIF3  PIF5  HAT5  SMZ  SEP3^b^ | ARR10  HMG-1  TGA1A  bZIP910  PI | ARR10  HMG-1  TGA1A  PIF3  PIF5  HAT5  ABI3 | HMG-1  ARR10  TGA1A  PIF3  PIF5  HAT5  ABI3  PI |  |  |

Relative profile score threshold = 95%

^a^ No significant motif was found.

^b^ Organ-specific motif exits in MADS-box genes.

**Table S4 Tissue-related coding motifs analysis of *Oncidum* gower ramsey MADS-box genes**

| **Class** | **Sepal** | **Petal** | **Lip** | **Stamen** | **Carpel** | **Class-unique  motifs** | **Essential for each class and tissue** |
| --- | --- | --- | --- | --- | --- | --- | --- |
| **A** |  |  |  | HMG-1, bZIP910, TGA1A, ARR10, PI |  | PIF5 | Dof2  Dof3  PBF  MNB1A |
| **AGL6** | HMG-1, ARR10, TGA1A | | | | | PIF4, MYC2, MYC3, MYC4, TGA1A |  |
| **OMADS1**  **(lip-specific)** |  |  | HMG-1, ARR10, SEP3 |  |  | ND^a^ |  |
| **B12** | ARR10 | |  |  |  | ND^a^ |  |
| **B34** |  | HMG-1, ARR10, TGA1, TGA1A, bZIP910, SMZ | |  |  | SMZ, ABI3 |  |
| **BPI** | ARR10 | | | | | ARR10 |  |
| **C** |  |  |  |  | ARR10, HMG-1, ABI3 | ND^a^ |  |
| **D** |  |  |  |  |  | HAT5 |  |
| **E** | HMG-1, ARR10, PIF3, PIF5, HAT5 | | |  | HMG-1, ARR10, PIF3, PIF5, HAT5 | ERF1 |  |
| **Tissue-related**  **motifs** | ARR10  HMG-1  PIF4  MYC2  MYC3  MYC4  TGA1A  SMZ  ABI3  ERF1 | ARR10  HMG-1  PIF4  MYC2  MYC3  MYC4  TGA1A  SMZ  ABI3  ERF1 | HMG-1  ARR10  PIF4  PIF5  MYC2  MYC3  MYC4  TGA1A  SMZ  ABI3  ERF1 | ARR10  HMG-1  PIF4  MYC2  MYC3  MYC4  TGA1A | HMG-1  ARR10  PIF4  PIF5  MYC2  MYC3  MYC4  TGA1A  ERF1  HAT5^b^ |  |  |

Relative profile score threshold = 95%

^a^ No significant motif was found.

^b^ Organ-specific motif exits in MADS-box genes.

**Datasets**

**Training dataset of class A**

>AT1G26310.1_CAL

MGRGRVELKRIENKINRQVTFSKRRTGLLKKAQEISVLCDAEVSLIVFSHKGKLFEYSSESCMEKVLERYERYSYAERQLIAPDSHVNAQTNWSMEYSRLKAKIELLERNQRHYLGEELEPMSLKDLQNLEQQLETALKHIRSRKNQLMNESLNHLQRKEKEIQEENSMLTKQIKERENILRTKQTQCEQLNRSVDDVPQPQPFQHPHLYMIAHQTSPFLNMGGLYQEEDQTAMRRNNLDLTLEPIYNYLGCYAA

>AT1G69120.1_AP1

MGRGRVQLKRIENKINRQVTFSKRRAGLLKKAHEISVLCDAEVALVVFSHKGKLFEYSTDSCMEKILERYERYSYAERQLIAPESDVNTNWSMEYNRLKAKIELLERNQRHYLGEDLQAMSPKELQNLEQQLDTALKHIRTRKNQLMYESINELQKKEKAIQEQNSMLSKQIKEREKILRAQQEQWDQQNQGHNMPPPLPPQQHQIQHPYMLSHQPSPFLNMGGLYQEDDPMAMRRNDLELTLEPVYNCNLGCFAA

>AT3G30260.1_AGL79

MGRGRVQLRRIENKIRRQVTFSKRRTGLVKKAQEISVLCDAEVALIVFSPKGKLFEYSAGSSMERILDRYERSAYAGQDIPTPNLDSQGECSTECSKLLRMIDVLQRSLRHLRGEEVDGLSIRDLQGVEMQLDTALKKTRSRKNQLMVESIAQLQKKEKELKELKKQLTKKAGEREDFQTQNLSHDLASLATPPFESPHELRRTISPPPPPLSSGDTSQRDGVGEVAAGTLIRRTNATLPHWMPQLTGE

>AT5G60910.1_AGL8

MGRGRVQLKRIENKINRQVTFSKRRSGLLKKAHEISVLCDAEVALIVFSSKGKLFEYSTDSCMERILERYDRYLYSDKQLVGRDVSQSENWVLEHAKLKARVEVLEKNKRNFMGEDLDSLSLKELQSLEHQLDAAIKSIRSRKNQAMFESISALQKKDKALQDHNNSLLKKIKEREKKTGQQEGQLVQCSNSSSVLLPQYCVTSSRDGFVERVGGENGGASSLTEPNSLLPAWMLRPTTTNE

>LOC_Os12g31748.2

MGRGKVQVRRIENEVSRQVTFSKRRPGLLKKAHEIAVLCDVDVAAIVFSAKGNLFHYASSHTTMERILEKYDRHELLSEGNNVIEEFPELEGSMSYDHIKLRGRIEALKKSQRNLMGQELDSLTLQDIQQLENQIDTSLNNIRSRKNNLLLKSIAELRQKEKLLMEKNTILEKKITELETLHTCIRASPTKAAAPPACNTADAFVPNLNICCGDSGEPETVTAPLGWTSSNNGLPWWMLQSSSNGKS

>LOC_Os07g41370.1

MGRGPVQLRRIENKINRQVTFSKRRNGLLKKAHEISVLCDADVALIVFSTKGKLYEFSSHSSMEGILERYQRYSFDERAVLEPNTEDQENWGDEYGILKSKLDALQKSQRQLLGEQLDTLTIKELQQLEHQLEYSLKHIRSKKNQLLFESISELQKKEKSLKNQNNVLQKLMETEKEKNNAIINTNREEQNGATPSTSSPTPVTAPDPIPTTNNSQSQPRGSGESEAQPSPAQAGNSKLPPWMLRTSHT

>LOC_Os03g54160.1

MGRGKVQLKRIENKINRQVTFSKRRSGLLKKANEISVLCDAEVALIIFSTKGKLYEYATDSCMDKILERYERYSYAEKVLISAESDTQGNWCHEYRKLKAKVETIQKCQKHLMGEDLESLNLKELQQLEQQLENSLKHIRSRKSQLMLESINELQRKEKSLQEENKVLQKENPCSFLQLVEKQKVQKQQVQWDQTQPQTSSSSSSFMMREALPTTNISNYPAAAGERIEDVAAGQPQHVRIGLPPWMLSHING

>LOC_Os07g01820.1

MGRGKVQLKRIENKINRQVTFSKRRNGLLKKAHEISVLCDAEVAAIVFSPKGKLYEYATDSRMDKILERYERYSYAEKALISAESESEITLPQLTTCTASRSTHGICFQYCLMSKTLGNWCHEYRKLKAKIETIQKCHKHLMGEDLESLNLKELQQLEQQLESSLKHIISRKSHLMLESISELQKKERSLQEENKALQKELVERQKNVRGQQQVGQWDQTQVQAQAQAQPQAQTSSSSSSMLRDQQALLPPQNICYPPVMMGERNDAAAAAAVAAQGQVQLRIGGLPPWMLSHLNA

>Pha_AP1-related_13

MGRGRVQLKRIENKINQQVTFSKRRSGLLKKAHEISVLCDADVALIIFSNKGKLCEYSTDSSMEKILERYEHYSYTERALYSNEDNPQADWRLEYNKMKAKVESLQKSQRHLMGEQLDYLSIKELQHLEQQLESSLKHIRSRKTQLMVDSISELQKKEKLLLEQNKTLQDMAKAKAKALVQNAAWEQQNKSQYSSEPPHAVISDSVPTPTSRTFQTRANGEESPQPQLRLGNSLLPPWMLSHVNG

>Pha_AP1-related_11

MGRGKVQLKRIENKINRQVTFSKRRSGLLKKAHEISVPCDAEVALVIFSAKGKLYGYSTDSCMDRILDRYERYCYAEKALQITEPESQGDICNEYGKLKNKIEALQKSRSHLMGEQLDSLSIKELQHLEQQLETALNHIRTQRIQLLLNCITEFQRKEKSLLEHNSLLEAKLCSFQLDPQITETATQNPNRKQQKQDQVNSSPSPFLPPNHLPTLNLGTYPASDGEEAEDPTLLQMNSISLPPWMLRSST

>Dendrobium_Thong-IN_DOMADS2

MGRGRVQLKRIENKINRQVTFSKRRSGLLKKAHEISVLCDAEVALIVFSNKGKLYEYSTDSSMEKILERYERYSYAERALFSNEANPQADWRLEYNKLKARVESLQKSQRHLMGEQLDSLSIKELQRLEQQLESSLKFIRSRKTQLILHSISELQKMEKILLEQNKTLEKEIIAKEKAKALVQHAPWEKQNQSQYSSALPPVISDSVPTPTSRTFQARANEEESPQPQLRVSNTLLPPWMLSHMNGQ

>Dendrobium_MADS-box_1

MGRGRVQLKRIENKINRQVTFSKRRSGLLKKAHEISVLCDAEVAVIVFSNKGKLYEFSTDSSMEKILERYERYSYAERALFSNEANPQADWHLEYHKLKARVESLQKSQRHLMGEQLDSLSIKELQHLEQQLESSMKHIRSRKTQLILDSISELQKKEKILLEQNKTLEKEIIAKEKAKALTQIAPWEKQNLSQYSSAPLHVISDSVPTPTSRTFQAIANEEESPQAQLRVSNTLLPPWMLGHMNG

>Cymbidium_faberi_MADS1

MGRGRVQLKRIENKINRQVTFSKRRSGLLKKAHEISVLCDAEVALIVFSNKGKLYEYSTEASMEKILERYERHSYAEKALFSNEANLQADWRLEYNKLKARVESLQKSKRHLMGEQLDSLSTKELQHLEQQLESSLKHIRSRKNQLMLDSISELQKKEKLLLDQNKTLEKEIMAKEKAKALVQNAPWEKQNQCQYSSAPSHAVISNFGSTPASRTLRARASEEESPQPQLRLGNTLLPPWMLTHMNG

**Training dataset of class B12**

>Pha_MADS5

MGRGKIEIKKIENPTSRQVTYSKRRLGIMKKAEELTVLCDAQLSLIIFSSSGKLADFCSPSTDVKDIVERYQNVTGIDIWDAQYQRMQNTLRNLREINRNLQKEIRQRKGENLEGLGVKELRGLEQKLEESVKIVRQRKYHVIATQTDTCRKKLKSSRQIYRALTHELQKLDEENQPCSFLVEDLSCIYDSSISMANRLHRSEPNVQKVVRECHEFGFD

>Pha_AP3-like_17

MGRGKIEIKKIENPTNRQVTYSKRRVGILKKAKELTVLCDAQVSLIMFSSTGKLADYCSPSTDIKGIYERYQVVTGMDLWNAQYERMQNTLKHLNEINQNLRKEIRRRKGEELEGMDIKQLRGLEQTLEESLRIVRHRKYHVIATQTDTYKKKLKSTRETYRALIHELDMKEENPNYGFNVENQSRIYENSIPMVNECPQMFSFRVVHPNQPNLLGLGYESHDLSLA

>Pha_MADS2

MGRGKIEIKKIENPTNRQVTYSKRRVGILKKAKELTVLCDAQVSLIMFSSTGKLADYCSPSTDIKGIYERYQVVTGMDLWNAQYERMQNTLKHLNEINQNLRKEIRRRKGEELEGMDIKQLRGLEQTLEESLRIVRHRKYHVIATQTDTYKKKLKSTRETYRALIHELDMKEENPNYGFNVENQSRIYENSIPMVNECPQMFSFRVVHPNQPNLLGLGYESHDLSLA

>Paphiopedilum_AP3-3

MGRGKIEIKKIENPTNGQVTYSKRRLGIMKKAKELSVLCDAQLSLIMFSNTGKLADYCSPSTDVKGIFERYQVVTGIDLWNVQYERMRRTLKHLEEINQSLRKEIMQRSGEGLEGMNIEELRGLEQTLDDSLRIVRQRKYHVIATQTDTYKKKLKSTRESCRLLMQELEMKDEHSHYDFVAEEHRRICENPIPMVNGAPPMYAFRVVQQIQPNLMDMSYDHSHDL

>Cymbidium_hybrid_cultivar_MADS2

MGRGKIEIKKIENPTNRQVTYSKRRVGILKKAKELTVLCDAQVSLIMFSSTGKLADYCSPSTDIKGIYERYQVVTGMDLWNAQYERMQNTLKHLNEINQNLRKEIRQRKGEELEGMDIKELRGLEQTLEESLRIVRQRKYHVIATQTDTYKKKLKSTRETYRALIKELEMKDENPNYGFSAENHSRIYENSIPMVNTECPQMFSFRVVQPNQPNLLGLGYESHDLSLA

>Cymbidium_hybrid_cultivar_MADS1

MGRGKIEIKKIENPTNRQVTYSKRRVGILKKAKELTVLCDAQVSLIMFSTTGKLADYCSPSTDIKGIYERYQIVTGMDLWNAQYERMQNTLNHLKEINQNLRKEIRQRNGEELEGLDIKELRGLEQTLEESIRIVRQRKYHVIATQTDTYKKKLKSTRETYRALIQELEMKDENPNYNFSAENHSRVYQNSIPMATECPQMFSFRVVQPTQPNLLGLGYESHDLSLA

>Dendrobium_AP3

MGRGKIEIKKIENPTNRQVTYSKRRVGILKKAKELTVLCDAQVSLIMFSSTGKLADYCSPVTDIKGIYERYQVVTGMDLWNAQYERMQNTLKHLNEINQNLRKEIRQRKGEELEGMEIKELRGLEQTLEESLRIVRQRKYHVIATQTDTYKKKLKSTRETYRALVHELEMKDDNPNYAFSAENHNRVYENSIPMVNDCPQLFSFRVVQPIQPNLLGIGYESHDLSLA

>Dendrobium_AP3-like_A

MGRGKIEIKKIENPTNRQVTYSKRRVGILKKAKELTVLCDAQVSLIMFSSTGKLADYCSPSTDIKGVYERYQVVTGIDLWNAQYERMQNTLKHLNEINQNLRKEIRQRKGEELEGMEIKELRGLEQTLEESLRIVRQRKYHVIATQTDTYKRKLKSTRETYRALVNELEMKDDNPNYAFSAENHSRVYENSIPMVNNDCPQMFSFRVVQPIQPNFLGIGYESHDLSLA

>Dendrobium_AP3-like_2

MGRGKIEIKKIENSTSRQVTYSKRRVGILKKAKELTVLCDAQVSLIMFSSTGKLADYCSPSADIKGIYERYQVVTGMDLWNAQYERMQNTLKHLSEINQNLRKEIRQRKGEELEGMEIKELRGLEQTLEESLRIVRQRKYHVIATQTDTYKKKFKSTKEAYRALVQQLEMKDENPHYAFSAENHNIVYENSIPMVNDCPQMFSFRVVQPMQPNLLGIGFESHDLSLA

**Training dataset of class B34**

>AT3G54340.1_AP3

MARGKIQIKRIENQTNRQVTYSKRRNGLFKKAHELTVLCDARVSIIMFSSSNKLHEYISPNTTTKEIVDLYQTISDVDVWATQYERMQETKRKLLETNRNLRTQIKQRLGECLDELDIQELRRLEDEMENTFKLVRERKFKSLGNQIETTKKKNKSQQDIQKNLIHELELRAEDPHYGLVDNGGDYDSVLGYQIEGSRAYALRFHQNHHHYYPNHGLHAPSASDIITFHLLE

>LOC_Os06g49840.1

MGRGKIEIKRIENATNRQVTYSKRRTGIMKKARELTVLCDAQVAIIMFSSTGKYHEFCSPSTDIKGIFDRYQQAIGTSLWIEQYENMQRTLSHLKDINRNLRTEIRQRMGEDLDGLEFDELRGLEQNVDAALKEVRHRKYHVITTQTETYKKKVKHSYEAYETLQQELGLREEPAFGFVDNTGGGWDGGAGAGAAADMFAFRVVPSQPNLHGMAYGGNHDLRLG

>Pha_MADS3

MGRGKIEIKKIENPTNRQVTYSKRRAGIMKKASELTVLCDAQLSLVMFSSTGKFSEYCSPTTDTKSVYDRYQQVSGINLWSEQYEKMQNTLNHLKEINHNLRREIRQRMGEDLEGLEIKELRGLEQNMDEALKLVRNRKYHVISTQTDTFKKKLKNSQETHRNLLRELETEHAVYYVDDDPNNYDGALALGNGASYLYSFRTQPSQPNLQGVGYVPHDLRLA

>Dendrobium_AP3-like_1

MGRGKIEIKKIENPTNRQVTYSKRRAGIMKKANELTVLCDAQLSLVMFSSTGKFSEYCSPSTDTKSIYDRYQQLSGINLWSAQYEKMQNTLNQLKEINHNLRREIRQRMGEDLDGLEIKELRGLEQNMDEALKLVRNRKYHVISTQTDTYKKKLKNSQETHRNLLRELETDHAVYYVDDDPSNYDGALALGNGASYLYSFRSQPSQPNLQGMGYGPHDLRLA

>Dendrobium_AP3-like_AP3B

MGRGKIEIKKIEYPTNRQVTYSKRRAGIMKKANELTVLCDAQLSLVMFSSTGKFSEYCSPSTDTKSIYDRYQQLSGINLWSAQYEKMQNTLNHLKEINHNLRREIRQRMGEDLDGLEIKELRGLEQNMDEALKLVRNRKYHVISTQTDTYKKKLKNSQETHRNLLRELETEHAVYYVDDDPSNYDGALALGNGASYLYSYRTQPSQPNLQGMGYGPHDLRLA

>Paphiopedilum_AP3-2

MGRGKIEIKKIENPTNRQVTYSKRRAGIMKKARELTVLCDAEVSLIMFSSTGKFSEYCSPSSDAKKVFDRYQQVSGINLWSVQYEKMQTTLNHLKEINHSLRREIRQRMGEDLEGLDIKELRGLEQNMDEALKLVRNRKYHVISTQTDTYKKKLKNSQETHRNLLRELEIIEDHPAFGYVDDDPSNYEGTLALANGSSHMFAFRVQPSQPNLHGMGFGSH

>Pha_MADS4

MGRGKIEIKKIENPTNRQVTYSKRRAGIMKKAREITVLCDAEVSLIMFSSTGKFSEYCSPSTETKKVFERYQQVSGINLWSSQYEKMLNTLNHSKEINRNLRREVRQRMGEDLEGLDIKELRGLEQNIDEALKLVRNRKYHVISTQTDTYKKKLKNSQETHRNLMHELEIVEDHPVYGFHEDSSNYEGVLALANDGSHMYAFRVQPNQQNLQGTGYSSHDLRLA

>Paphiopedilum_DEF

MGRGKIEIKRIENPTSRQVTYSKRRAGIMKRASELSVLCDAELSLVMFSSTGRFSEYCSPSTDAKSMYDRYQQATGIDLWSTQYEKMQNMLSHLKEVNHNLRREISQRMGEDLDGMDIKELRGLEQNIDEALNLVRSRKYHVISTQTDTYKKKLKNSQETHKNLIRELEMEEHAVFGYVDDDQCNNDGGLALVNEASYNFSFRAQPSPANFLGMGYGSHDLRLA

>Paphiopedilum_AP3-1

MGRGKIEEKRIENPTSRQVTYSKGRAGIMKKASELSVLCDAELSLVMFSSTGRFSEYCSPSTDAKSMYDRYQQATGIDLWSTQYEKMQNMLSHLKEVNRSLRREISQRMGEDLDGMDIKELRGLEQNIDEALNLVRSRKYHVISTQTDTYKKKLKNSQETHKNLIRGLEMEEHAVFGYVDDDQCNNDGALALVNEASYNFSFRAQPSAANFLGMGYGSHDL

>Dendrobium_AP3-related_MADS4

MGRGKIEIKKIENPTNRQVTYSKRRAGIMKKAKEITVLCDAEVSLIMFSSTGKFSEYCSPSMETEKIFDRYQQLSGINLWSAHYEKMQNTLNHLKEINHNLRREVRQRMGEDLEGLDIKELRGLEQNMDEALKLVRNRKCHVISTQTDTYKKKLKNSQETHRNLMHEMEVVEDHTVFGFDDDSSNYEGVLALANGGSHMYAFRVQPSQPNLHEMGYGPHDLRLA

**Training dataset of class BPI**

>AT5G20240.1_PI

MGRGKIEIKRIENANNRVVTFSKRRNGLVKKAKEITVLCDAKVALIIFASNGKMIDYCCPSMDLGAMLDQYQKLSGKKLWDAKHENLSNEIDRIKKENDSLQLELRHLKGEDIQSLNLKNLMAVEHAIEHGLDKVRDHQMEILISKRRNEKMMAEEQRQLTFQLQQQEMAIASNARGMMMRDHDGQFGYRVQPIQPNLQEKIMSLVID

>Dendrobium_PI-related

MGRGKIEIKRIENSTNRQVTFSKRRNGIMKKAKEISVLCDAQVSLVIFSSLGKMFEYCSPSTSLSKMLEKYQQNSGKKLWDAKHENLSAEIDRIKKENDNMQIELRHLKGEDLNSLNPKELIPIEEALQNGLTGVRDKQMDFLKMLKKNERMLEEENKRLTYLLHHQQLAMEGSMRELDIGYHQKDREYAAQMPLTFRVQPIQPNLQGNK

>Paphiopedilum_PI

MGRGKIEIKRIENSTNRQVTFSKRRNGIMKKAKEISVLCDAQVSLVIFSSLGKMSEYCSPATTLSKMLEKYQQNSGKKLWDAKHENLSAEIDRIKKENDNMQIELRHLKGEDLNSLNPKELIPIEEALQNGLTSVRDKQMNFLKLLKKNERMLEDENKRLTYLLHHQELAMEGSMRELDISYHQKDREYGNQMPMAFRVQPIQPNLQGN

>Paphiopedilum_GLO

MGRGKIEIKRIENSTNRQVTFSRRRNGIMKKAKEISVLCDAQVSLVIFSSLGKMSEYCSPSTTLSKMLEKYQQNSGKKLWDAKHENLSAEIDRIKKENDNMQIELRHMKGEDLNSLNPKELIPIEEALQNGLTSVRDKQMNFLKMLKRNERMLEDENKRLTYLLHHQELAMEGSMRELDISYHQKDREYGNQMPMAFRVQPIQPNLQGNK

>Pha_MADS6

MGRGKIEIKRIENSTNRQVTFSKRRNGIMKKAKEISVLCDAQVSLVIFSSLGKMFEYCSPSTTLSKMLEKYQQNSGKKLWDAKHENLSAEIDRIKKENDNMQIELRHLKGEDLNSLNPKELIPIEEALQNGLTSVRDKQMDYLKMLKKNERMLEDENKRLTYLLHQQQMAMEGSMRELDIGYHHKDREYAAQMPMTFRVQPIQPNLQGNK

>Pha_PI-like_9

MGRGKIEIKRIENSTNRQVTFSKRRNGIMKKAKEISVLCDAQVSLVIFSSLGKMFEYCSPSTTLSKMLEKYQQNSGKKLWDAKHENLSAEIDRIKKENDNMQIELRHLKGEDLNSLNPKELIPIEEALQNGLTSVRDKQMDYLKMLKKNERMLEDENKRLTYLLHQQQMAMEGSMRELDIGYHHKDREYAAQMPMTFRVQPIQPNLQGNK

>Pha_PI-like_10

MGRGKIEIKRIENSTNRQVTFSKRRNGIMKKAKEISVLCDAQVSLVIFSSLGKMFEYCSPSTTLSKMLEKYQQNSGKKLWDAKHENLSAEIDRIKKENDNMQIELRHYEGEDLNSLNPKELIPIEEALQNGLTSVRDKQMDYLKMLKKNERMLEDENKRLTYLLHQQQMAMEGSMRELDIGYHHKDREYAAQMPMTFRVQPIQPNLQGNK

>Pha_PI-like_15

MGRGKIEIKRIENSTNRQVTFSKRRNGIMKKAKEISVLCDAQVSLVIFSSLGKMFEYCSPSTTLSKMLEKYQQNSGKKLWDAKHENLSAEIDRIKKENDNMQIELRFSWVLSRHLKGEDLNSLNPKELIPIEEALQNGLTSVRDKQMDYLKMLKKNERMLEDENKRLTYLLHQQQMAMEGSMRELDIGYHHKDREYAAQMPMTFRVQPIQPNLQGNK

>LOC_Os01g66030.1

MGRGKIEIKRIENSTNRQVTFSKRRSGILKKAREISVLCDAEVGVVIFSSAGKLYDYCSPKTSLSRILEKYQTNSGKILWDEKHKSLSAEIDRIKKENDNMQIELRHLKGEDLNSLQPKELIMIEEALDNGIVNVNDKLMDHWERHVRTDKMLEDENKLLAFKLHQQDIALSGSMRDLELGYHPDRDFAAQMPITFRVQPSHPNLQENN

>LOC_Os05g34940.1

MGRGKIEIKRIENSTNRQVTFSKRRAGILKKAREIGVLCDAEVGVVIFSSAGKLSDYCTPKTTSVFPPLSRILEKYQTNSGKILWDEKHKSLSAEIDRVKKENDNMQIELRHMKGEDLNSLQPKELIAIEEALNNGQANLRDKMMDHWRMHKRNEKMLEDEHKMLAFRVHQQEVELSGGIRELELGYHHDDRDFAASMPFTFRVQPSHPNLQQEK

**Training dataset of class C**

>AT4G18960.1_AG

TAYQSELGGDSSPLRKSGRGKIEIKRIENTTNRQVTFCKRRNGLLKKAYELSVLCDAEVALIVFSSRGRLYEYSNNSVKGTIERYKKAISDNSNTGSVAEINAQYYQQESAKLRQQIISIQNSNRQLMGETIGSMSPKELRNLEGRLERSITRIRSKKNELLFSEIDYMQKREVDLHNDNQILRAKIAENERNNPSISLMPGGSNYEQLMPPPQTQSQPFDSRNYFQVAALQPNNHHYSSAGRQDQTALQLV

>LOC_Os01g10504.1

MMNMMTDLSCGPSSMTELTAAAAPAGSGSSAAVAAGSSEKMGRGKIEIKRIENTTNRQVTFCKRRNGLLKKAYELSVLCDAEVALIVFSSRGRLYEYANNSVKSTVERYKKANSDTSNSGTVAEVNAQHYQQESSKLRQQISSLQNANSRTIVGDSINTMSLRDLKQVENRLEKGIAKIRARKNELLYAEVEYMQKREVELQNDNMYLRSKVVENERGQQPLNMMGAASTSEYDHMVNNPYDSRNFLQVNIMQQPQHYAHQLQPTTLQLGSRPSISFGVDTVRTHVR

>LOC_Os01g66290.1

MGRGKIEIKRIENKTSRQVTFCKRRNGLLKKAYELAILCDAEIALIVFSSRGRLYEFSNVNSTRSTIERYKKASASTSGSAPVIDVNSHQYFQQEAAKMRHQIQTLQNANRHLIGESIGNMTAKELKSLENRLEKGISRIRSKKHELLFSEIEYMQKREADLQNENMFLRAKVAEAERAEHDDQQAAEDDEMAPAPAVGGGSSSGTELEALPATFDTREYYQPAPPVSMLAAAAAAAAAQYSSDHHQTALHLGYFKVDSGKGGLL

>Pha_MADS1

MDSSSMEPKEKMGRGKIEIKRIENTTNRQVTFCKRRNGLLKKAYELSVLCDAEVALIIFSTRGRLYEYANNSVKGTIERYKKASTDNSNTGSISEANSQYYQQEATKLRQQITNLQNSNRNLLGDALTTMSLRDLKQLETRLEKGINKIRAKKNELLHAEIDYMQKREMELQTDNMFLRNKISDNERAQQQHQHMSILPSTSTEYEVMPPFDSRSFLHVNLMDPNDRYSHQQQTALQLG

>Pha_AG1

MDSSSMEPKEKMGRGKIEIKRIENTTNRQVTFCKRRNGLLKKAYELSVLCDAEVALIIFSTRGRLYEYANNSVKGTIERYKKASTDNSNTGSISEANSQYYQQEATKLRQQITNLQNSNRNLLGDALTTMSLRDLKQLETRLEKGINKIRAKKNELLHAEIDYMQKREMELQTDNMFLRNKISDNERAQQQHQHMSILPSTSTEYEVMPPFDSRSFLHVNLMDPNDRYSHQQQTALQLG

>Dendrobium_MADS-box_2

MGRGKIEIKRIENTTNRQVTFCKRRNGLLKKAYELSVLCDAEIALIVFSTRGRLYEYSNHSIKATIEKYKKACADSSNPGSLVEVNSQQYYQQESAKLRHQIQLLQNSNRHLMGEGLSSLTLKELKQLENRLERGITRVRSKKHELLFAEIEYMQKREVELQNDNMYLRAKINDNERAEHANIVQAGTDFDTLPNFDSRNYYHLNILETAPHYSHHQDQTALHLGYETKADHSA

>Dendrobium_SEEDSTICK-like_AG2

MGRGKIEIKRIENTTNRQVTFCKRRNGLLKKAYELSVLCDAEIALIVFSTRGRLYEYSNHSIKATIEKYKKACADSSNPGSLVEVNSQQYYQQESAKLRHQIQLLQNSNRHLMGEGLSSLTLKELKQLENRLERGITRVRSKKHELLFAEIEYMQKREVELQNDNMYLRAKINDNERAEQANIVQAGADFDTLPNFDSRNYYQVNILETAAHYSHHQDQTALHLGYETKADHSA

>Dendrobium_crumenatum_AG2

MGRGKIEIKRIENTTNRQVTFCKRRNGLLKKAYELSVLCDAEVALIVFSSRGRLYEYSNSSTNSTIERYKKAITNSSNSVVEVNSQQYYQQEAAKLRHQIQILHNTNRHPMGEGLTSLSIKELKQLESRLERGITRIRSKKHEMLFAEIEFMQKREEDLQNENMYLRAKITENERQTNIDTTASALDTLSTFDSRNYYPVNMLEAAAHYHNQDQTALHLGYDN

>Dendrobium_crumenatum_AG1

MMEPKEKMGRGKIEIKRIENTTNRQVTFCKRRNGLLKKAYELSVLCDAEVALVIFSSRGRLYEYANNSVKGTIERYKKASADNSNSGSISETNAQYYLQEASKLRQQITNLQNSNRNLMGEALSTMSLRDLKQLETRLEKGINKIRSKKNELLYAEIEYMQKREMELQNDNMYLRNKIADNERTQQQHHINMVPSTSTEYEVMPPFDSRNFLQVNLMDPSHHYSLQQQTALQVG

>Cymbidium_ensifolium_MADS1

MEPKEKMGRGKIEIKRIENTTNRQVTFCKRRNGLLKKAYELSVLCDAEVALVIFSSRGRLYEYANNSVKGTIDRYKKACTDNSSTGSISEANSQYYQQEATKLRQQITNLQNSNRNLLGDALTTMSLRDLKQLETRLEKGISKIRSKKNELLHAEIDYMQKREMDLQTDNMYLRSKIADNERAQQHQHMSILPSTSTEYEVMPPFDSRSFLQVNLLDPSDHYSHQQQTALQLG

>Dendrobium_thyrsiflorum_AG1

MEPKEKMGRGKIEIKRIENTTNRQVTFCKRRNGLLKKAYELSVLCDAEVALIIFSSRGRLYEYANNSVKGTIDRYKKANSDNSNSGSISEANSQYYQQEATKLRQQITNLQNNRNLLGDALTTMSLRDLKQLETRLEKGINKIRSKKNELLHAEIDYMQKREMDLQTDNMYLRNKIADNERAQQHQHMNILPSTSAEYEVMPPFDSRSFLQVNLLDPNDHYAHQQQTALQLG

>Cymbidium_ensifolium_MADS2

MMEPKEKMGRGKIEIKRIENTTNRQVTFCKRRNGLLKKAYELSVLCDAEVALVIFSTRGRLYEYANNSVKATIEKYKKACSDNSNSGTISETNAQYYMQEASKLRQQITNLQNSNRNLMGEALSTMSLRDLKQLETRLEKGINKIRSKKNELLYAEIEYMQKREMELQNDNMYLRNKIAENERTQQQPHINMVPSTSTEYEVMPPFDSRNFLQVNLMDPSHHYSLQQQTALQLG

>LOC_Os05g11414.1

MHIYKEQEAEPSTGLMMPEPAPVASPGSGGSGGSGSVGAEKIGSRGKIEIKRIENTTNRQVTFCKRRSGLLKKAYELSVLCDAEVALVVFSSRGRLYEYSNNSVKETIERYKKANSDTSNASTVAEINAQHYQQEAAKLKQQITNLQNSNRTLVGDNITTMNHRELKQLEGRLDKGLGKIRARKNELLCAEIEYMQRRETELQNDNMYLKSKVAESERGLQTVNMMGSASTSEYVQNMIHYDPRNFLQFNIMHQPQYYPEQEDRKAFMSGKKYSQCNIVRVHSSTNEI

>LMADS_10

MGRGKIEIKRIENTTNRQVTFCKRRNGLLKKAYELSVLCDAEVALIVFSTRGRLYEYANNSVEATIERYKKASTDISNTRSVSEANAQYYQQESTKLRQQINSLQNSNRNLLGESLSNMNLRDLKQLENRLEKAINKIRTKKNELLYAEIEYMQKREMELQSDNMYLRNKVAENEREQQQQMNMMPSTSEYEAMPHFDSRNFLQVNIVDPNQHYSCQQQTALQLG

**Training dataset of class D**

>AT4G09960.1_STK

MGRGKIEIKRIENSTNRQVTFCKRRNGLLKKAYELSVLCDAEVALIVFSTRGRLYEYANNNIRSTIERYKKACSDSTNTSTVQEINAAYYQQESAKLRQQIQTIQNSNRNLMGDSLSSLSVKELKQVENRLEKAISRIRSKKHELLLVEIENAQKREIELDNENIYLRTKVAEVERYQQHHHQMVSGSEINAIEALASRNYFAHSIMTAGSGSGNGGSYSDPDKKILHLG

>AT2G42830.1_SHP2

MEGGASNEVAESSKKIGRGKIEIKRIENTTNRQVTFCKRRNGLLKKAYELSVLCDAEVALVIFSTRGRLYEYANNSVRGTIERYKKACSDAVNPPTITEANTQYYQQEASKLRRQIRDIQNLNRHILGESLGSLNFKELKNLESRLEKGISRVRSKKHEMLVAEIEYMQKREIELQNDNMYLRSKITERTGLQQQESSVIHQGTVYESGVTSSHQSGQYNRNYIAVNLLEPNQNSSNQDQPPLQLV

>AT3G58780.1_SHP1

MEEGGSSHDAESSKKLGRGKIEIKRIENTTNRQVTFCKRRNGLLKKAYELSVLCDAEVALVIFSTRGRLYEYANNSVRGTIERYKKACSDAVNPPSVTEANTQYYQQEASKLRRQIRDIQNSNRHIVGESLGSLNFKELKNLEGRLEKGISRVRSKKNELLVAEIEYMQKREMELQHNNMYLRAKIAEGARLNPDQQESSVIQGTTVYESGVSSHDQSQHYNRNYIPVNLLEPNQQFSGQDQPPLQLV

>LMADS_2

MGRGKIEIKRIENTTNRQVTFCKRRNGLLKKAYELSVLCDAEVALIVFSSRGRVYEYSNNSIKQTIDRYKKACDSSNSNSLIQVNSQQYFQQESAKLRHQIQILTNANRHLVGEALSSLTVKELKQLENRLERGLTRIRSKKHELLFAEIEFSQKREVELQSDNMYLRAKIAENERTQAAIVQARAEFDALPTFDSRNFYQVNNMLEAPPHYHHQDQTALHLGYEAKDDSVP

>Pha_MADS7

MGRGKIEIKRIENTTNRQVTFCKRRNGLLKKAYELSVLCEAEIALIVFSSRGRVYEYANNSIKATIEKYKKTCAGSSNPGSLVEVNSHQYYQQESAKMRHQIQLLQNSNRHLMGDGLSSLNLKELKQLENRLERGITRVRSKKHELLFAEIEYMQKREVELQNDNMYLRAKIADNERAQQANIVQAGVDFESIPSFDSRNYYHINMLESASHYSHHQDQTALHLGYETKADQSA

>PhalAG2

IKRIENTTNRQVTFCKRRNGLLKKAYELSVLCEAEIALIVFSSRGRVYEYSNNSIKATIEKYKKTCAGSSNPGSLVEVNSHQYYQQESAKMRHQIQLLQNSNRHLMGDGLSSLNLKELKQLENRLERGITRVRSKKHELLFAEIEYMQKREVELQNDNMYLRAKIADNERAQQANIVQAGVDFESIPSFDSRNYYHINMLESASHYSHHQDQTALHLGYETKADQSA

>APMADS2

MGRGKIEIKRIENTTNRQVTFCKRRNGLLKKAYELSVLCDAEVALIVFSTRGRLYEYSNNSIKSTIERYKKACADSSNSTAVVEVNTQQYYQQEAAKLRHQIQSLQNSNRHLMGDSLSSLSIKELKQLENRLERGITRIRSKKHELLFAEIEYMQKREAELQNDNMYLRAKITDNERAHQVSVVQSGTEYDTLPTFDSRNYYTHVTMLEAAPHFSHHQDHTALHLGYETKADPTE

>HOMADS1

MGRGKIEIKRIENTTNRQVTFCKRRNGLLKKAYELSVLCDAEVALIVFSTRGRLYEYSNNSIKSTIERDKKACADSSSSSAVIEVNTQRYYQQEASKLRQQIQILQNANRHLMGESLDPLNVKELKQLETRLERGITRVRSKKHELLFAELEYMQKREVELQTDNMYLRAKIGENERAHQASVVQAGTEFDALPTFDSRNYYQVHMLQAASHYSHHQDQTALHLGYETKADPSA

**Training dataset of class E**

>AT5G15800.1_SEP1

MGRGRVELKRIENKINRQVTFAKRRNGLLKKAYELSVLCDAEVALIIFSNRGKLYEFCSSSNMLKTLDRYQKCSYGSIEVNNKPAKELENSYREYLKLKGRYENLQRQQRNLLGEDLGPLNSKELEQLERQLDGSLKQVRSIKTQYMLDQLSDLQNKEQMLLETNRALAMKLDDMIGVRSHHMGGGGGWEGGEQNVTYAHHQAQSQGLYQPLECNPTLQMGYDNPVCSEQITATTQAQAQQGNGYIPGWML

>AT3G02310.1_SEP2

MGRGRVELKRIENKINRQVTFAKRRNGLLKKAYELSVLCDAEVSLIVFSNRGKLYEFCSTSNMLKTLERYQKCSYGSIEVNNKPAKELENSYREYLKLKGRYENLQRQQRNLLGEDLGPLNSKELEQLERQLDGSLKQVRCIKTQYMLDQLSDLQGKEHILLDANRALSMKLEDMIGVRHHHIGGGWEGGDQQNIAYGHPQAHSQGLYQSLECDPTLQIGYSHPVCSEQMAVTVQGQSQQGNGYIPGWML

>AT1G24260.1_SEP3

MGRGRVELKRIENKINRQVTFAKRRNGLLKKAYELSVLCDAEVALIIFSNRGKLYEFCSSSSMLRTLERYQKCNYGAPEPNVPSREALAELSSQQEYLKLKERYDALQRTQRNLLGEDLGPLSTKELESLERQLDSSLKQIRALRTQFMLDQLNDLQSKERMLTETNKTLRLRLADGYQMPLQLNPNQEEVDHYGRHHHQQQQHSQAFFQPLECEPILQIGYQGQQDGMGAGPSVNNYMLGWLPYDTNSI

>AT2G03710.1_SEP4

MGRGKVELKRIENKINRQVTFAKRRNGLLKKAYELSVLCDAEIALLIFSNRGKLYEFCSSPSGMARTVDKYRKHSYATMDPNQSAKDLQDKYQDYLKLKSRVEILQHSQRHLLGEELSEMDVNELEHLERQVDASLRQIRSTKARSMLDQLSDLKTKEEMLLETNRDLRRKLEDSDAALTQSFWGSSAAEQQQQHQQQQQGMSSYQSNPPIQEAGFFKPLQGNVALQMSSHYNHNPANATNSATTSQNVNGFFPGWMV

>LOC_Os06g06750.1

MGRGKVELKRIENKISRQVTFAKRRNGLLKKAYELSVLCDAEVALIIFSTRGRLFEFSTSSCMYKTLERYRSCNYNLNSCEASAALETELSNYQEYLKLKTRVEFLQTTQRNLLGEDLVPLSLKELEQLENQIEISLMNIRSSKNQQLLDQVFELKRKEQQLQDANKDLKRKIQETSGENMLHISCQDVGPSGHASEANQEFLHHAICDPSLHIGYQAYMDHLNQ

>LOC_Os03g11614.1

MGRGKVELKRIENKISRQVTFAKRRNGLLKKAYELSLLCDAEVALIIFSGRGRLFEFSSSSCMYKTLERYRSCNYNSQDAAAPENEINYQEYLKLKTRVEFLQTTQRNILGEDLGPLSMKELEQLENQIEVSLKQIRSRKNQALLDQLFDLKSKEQQLQDLNKDLRKKLQETSAENVLHMSWQDGGGHSGSSTVLADQPHHHQGLLHPHPDQGDHSLQIGYHHPHAHHHQAYMDHLSNEAADMVAHHPNEHIPSGWI

>LOC_Os03g54170.1

MGRGKVVLQRIENKISRQVTFAKRRNGLLKKAYELSILCDAEVALVLFSHAGRLYQFSSSSNMLKTLERYQRYIYASQDAAAPTSDEMQNNYQEYVNLKAHVEILQQSQRNLLGEDLAPLATNELEQLESQVVRTLKQIRSRKTQVLLDELCDLKRKEQMLQDANRVLKRKLDEIDVEAAPPQPPWNGNCSNGHGGGGGVFSSEPPQPEHFFQALGLHAVDVNQPPAPPPGGYPPEWMA

>LOC_Os08g41950.1

MAEKKKKKKKKKPQSLLVLTSWRSIGMGRGRVELKRIENKINRQVTFAKRRNGLLKKAYELSVLCDAEVALIIFSNRGKLYEFCSTQSMTKTLEKYQKCSYAGPETAVQNRESEQLKASRNEYLKLKARVENLQRTQRQYYKSKHRLCLVRSKVWNLVKIRDDVTEKLCMYERNLLGEDLDSLGIKELESLEKQLDSSLKHVRTTRTKHLVDQLTELQRKEQMVSEANRCLRRKLEESNHVRGQQVWEQGCNLIGYERQPEVQQPLHGGNGFFHPLDAAGEPTLQIGYPAEHHEAMNSACMNTYMPPWLP

>LOC_Os09g32948.1

MGRGRVELKRIENKINRQVTFAKRRNGLLKKAYELSVLCDAEVALIIFSNRGKLYEFCSGQSMTRTLERYQKFSYGGPDTAIQNKENELVQSSRNEYLKLKARVENLQRTQRNLLGEDLGTLGIKELEQLEKQLDSSLRHIRSTRTQHMLDQLTDLQRREQMLCEANKCLRRKLEESNQLHGQVWEHGATLLGYERQSPHAVQQVPPHGGNGFFHSLEAAAEPTLQIGFTPEQMNNSCVTAFMPTWLP

>Dendrobium_SEP1

MGRGRVELKRIENKINRQVTFAKRRNGLLKKAYELSILCDAEVALIIFSNRGKLYEFCSSSSMLKTLERYQKCNYEGPETNIISRETQSSQQEYLKLKARVEALQRSQRNLLGEDLGPLSSKELEHLERQLDASLKQIRSTRTQFMLDQLADLQRREQMLCEANKALKRRFEESNQTAHQQVWDPSTTHAVGYGRQPAQHHGDAFYHPLECEPTLQIGYHSDITMAPTTAPNVSNYMPPGWLV

>Dendrobium_SEP3-like

MGRGRVEMKRIENKINRQVTFAKRRTGLLKKAYELSVLCDVGVALIIFSNRGKLYEFCSSRSMLKTLERYQKSNNGAPEMTMTSRETQSSQGEYLKLKAQVEALQRSQRNLMGEDLSPLGAKDLDQLEHQLEASLKQIRSTRMQYMLDQLCDLQQRELLLFETNKSLRTRLEEITQVSTQPFWDPNISQTLGYERRPDQLQGDDFYHPLEFEPTLQMGFPTTSLGGS

>Dendrobium_Thong-IN_otg7

MGRGRVEMKRIENKINRQVTFAKRRTGLLKKAYELSVLCDVEVALIIFSNRGKLYEFCSSRSMLKTLEKYQKCSDGAPEMTMTSRETQSSQVEYLKLKSQVEALQRSQRNLLGEDLNPLGGKDLDQLERQLEASLKQIISTRMQYMLDQLGDLQQRELLLFETNKSLGTRVSAL

>Dendrobium_Thong-IN_DOMADS1

MGRGRVEMKRIENKINRQVTFAKRRTGLLKKAYELSVLCDVEVALIIFSNRGKLYEFCSSRSMLKTLEKYQKCSDGAPEMTMTSRETQSSQVEYLKLKSQVEALQRSQRNLLGEDLNPLGGKDLDQLERQLEASLKQIISTRMQYMLDQLGDLQQRELLLFETNKSLGTRVSAL

>Dendrobium_Thong-IN_DOMADS3

MGRGRVELKRIENKINRQVTFAKRRNGLLKKAYELSVLCDAEVALIVFSNRGRLFEFCSSTSMTKTLERYQKCSYNASESAVPSKDAQNSYHEYLTLKAKVEYLQRSQGNLLGEDLIELSSKELDQLELQLEMSLKQIRSTKTQLMLDQLCDIKRKEQMLHEANRALSMKLKEDGPEIPLELSWPGGETNGSSERQQPQSDKFFQPLPCSNPSLQIGYSP

**Training dataset of class AGL6**

>AT2G45650.1_AGL6

MGRGRVEMKRIENKINRQVTFSKRRNGLLKKAYELSVLCDAEVALIIFSSRGKLYEFGSVGIESTIERYNRCYNCSLSNNKPEETTQSWCQEVTKLKSKYESLVRTNRNLLGEDLGEMGVKELQALERQLEAALTATRQRKTQVMMEEMEDLRKKERQLGDINKQLKIKFETEGHAFKTFQDLWANSAASVAGDPNNSEFPVEPSHPNVLDCNTEPFLQIGFQQHYYVQGEGSSVSKSNVAGETNFVQGWVL

>AT3G61120.1_AGL13

MGRGKVEVKRIENKITRQVTFSKRKSGLLKKAYELSVLCDAEVSLIIFSTGGKLYEFSNVGVGRTIERYYRCKDNLLDNDTLEDTQGLRQEVTKLKCKYESLLRTHRNLVGEDLEGMSIKELQTLERQLEGALSATRKQKTQVMMEQMEELRRKERELGDINNKLKLETEDHDFKGFQDLLLNPVLTAGCSTDFSLQSTHQNYISDCNLGYFLQIGFQQHYEQGEGSSVTKSNARSDAETNFVQ

>LOC_Os02g45770.1

MGRGRVELKRIENKINRQVTFSKRRNGLLKKAYELSVLCDAEVALIIFSSRGKLYEFGSAGITKTLERYQHCCYNAQDSNNALSETQSWYHEMSKLKAKFEALQRTQRHLLGEDLGPLSVKELQQLEKQLECALSQARQRKTQLMMEQVEELRRKERQLGEINRQLKHKLEVEGSTSNYRAMQQASWAQGAVVENGAAYVQPPPHSAAMDSEPTLQIGYPHQFVPAEANTIQRSTAPAGAENNFMLGWVL

>LOC_Os04g49150.1

MDRSEMGRGRVELKRIENKINRQVTFSKRRNGLLKKAYELSVLCDAEVALIIFSSRGKLYEFGSAGINKTLEKYNSCCYNAQGSNSALAGGEHQSWYQEMSRLKTKLECLQRSQRHMLGEDLGPLSIKELQQLEKQLEYSLSQARQRKTQIMMEQVDDLRRKERQLGELNKQLKNKLEAEADSSNCRSAIQDSWVHGTVVSGGRVLNAQPPPDIDCEPTLQIGYYQFVRPEAANPRSNGGGGDQNNNFVMGWPL

>Dendrobium_hybrid_cultivar_AGL6

MGRGRVELKRIENKINRQVTFSKRRNGIMKKAYELSVLCDAEIALIIFSSRGKLFEFGSPDITKTLERYQRCTFTPQTIHPNDHETLNWYQELSKLKAKYESLQRSQRHLLGEDLDMLSLKELQQLERQLESSLSQARQKRTQIMLNQMEELKKKERHLGDINKQLKHKLGADGGSMRALQGSWRPDAGASIDAFPNHSSNMDTEPTLHIGRYQQYVPSEATIPRNSGSGNGFMPGWAV

>Cymbidium_faberi_AGL6

MGRGRVELKRIENKINRQVTFSKRRNGLLKKAYELSVLCDAEVALIIFSSRGKLYEFGSAGTCKTLERYQRSCLNSQATNSIDRETQSWYQEVSKLKSKFESLQRSHRNLLGEDLGPLNVKELQQLERQPETALSQARQRKTQIMLDQMEELRKKERQLGEINKQLKMKLEAGGGSLRLMQGSWESDAVVEGNAFQMHPYQSSSLECEPTLHIGYHHYVPPETVIPRTPGVENNNFMLGWML

>Cymbidium_goeringii_AGL6

MGRGRVELKRIENKINRQVTFSKRRNGLLKKAYELSVLCDAEVALIIFSSRGKLYEFGSAGTCKTLERYQRSCLNSQATNSIDRETQSWYQEVSKLKSKFESLQRSHRNLLGEDLGPLNVKELQQLERQLETALSQARQRKTQMMLDQMEELRKKERQLGEINKQLKMKLEAGGGSLRLMQGSWESDAVVEGNAFQMHPYQSSSLECEPTLHIGYHHYVPPETVIPRTPGVENNNFMLGWML
